# Embodied cognition and urban design: Thoughts through epigenetic advances

**DOI:** 10.1192/j.eurpsy.2024.1299

**Published:** 2024-08-27

**Authors:** E. Abdelmoula, B. Abdelmoula, N. Bouayed Abdelmoula

**Affiliations:** ^1^LR AMC, Ecole Doctorale Sciences et Ingénierie Architecturales (ED-SIA), Tunis; ^2^Genomics of Signalopathies at the service of Precision Medicine - LR23ES07, Medical University of Sfax, Sfax, Tunisia

## Abstract

**Introduction:**

In the history of urban planning, the cognitive trend has been a well-established entity since the work of the American urban planner during the mid-’90s; Kevin Lynch. However, for a long time, urban planning has been deprived of the contribution of scientific knowledge from cognitive neurosciences, with a lack of operational recommendations for urban projects.

**Objectives:**

This study aims to reveal the role of embodiment theories in the revolution of urban design and urban projects through emerging findings in epigenetics and post-genomic biology.

**Methods:**

We conducted an exhaustive review of the scientific literature to establish the relationship between embodied cognition and urban design through advances in epigenetics as well as potential applications of such finding. Our inquiry was to find out whether there was a scientific way to measure and quantify the performance of urban spaces.

**Results:**

Our review revealed that, epigenetics and epigenomics have provided new explanations and perspectives to certain debates on the theory of embodied cognition and that of enaction. Epigenetic marks constitute a bodily memory that enables cognition to emerge as a function of the level of adaptation to the environment. In fact, embodiment refers to thoughts, emotions and behaviors based on sensory experiences and bodily positions, while the enaction is a way of conceiving cognition that focuses on the way in which human organisms and minds organize themselves in interaction with the environment.

**Conclusions:**

Cognition is the result of a level of adaptation to the environment determined by physiological parameters that confer possibilities of action depending on previous interactions with the environment. The regulation of epigenetic marks which are technically quantifiable is now recognized as the fundamental mechanism involved in the brain’s ability to create, dismantle or reorganize neural networks throughout life depending on various experiences including environmental ones.

**Disclosure of Interest:**

None Declared

